# Access to a tailored mobile application enhances medication adherence among young users of antidepressants

**DOI:** 10.3389/fphar.2024.1379700

**Published:** 2024-04-10

**Authors:** Rønnaug Eline Larsen, Kristine Hole, Maria Lie Selle, Cecilie Johannessen Landmark, Tonje Krogstad, Lene Berge Holm

**Affiliations:** ^1^ Department of Pharmacy, Oslo Metropolitan University, Oslo, Norway; ^2^ Center for Psychopharmacology, Diakonhjemmet Hospital, Oslo, Norway; ^3^ Health Services Research Unit, Akershus University Hospital, Lørenskog, Norway; ^4^ The National Centre for Epilepsy, Member of the ERN Network EpiCare, Oslo University Hospital, Oslo, Norway; ^5^ Section for Clinical Pharmacology, Department of Pharmacology, Oslo University Hospital, Oslo, Norway

**Keywords:** SSRI, SNRI, OMAS-37, antidepressants, medication adherence, mHealth, mobile apps

## Abstract

**Introduction:**

Patients’ adherence to antidepressants is generally reported to be poor. This study examined whether users of selective serotonin reuptake inhibitors (SSRIs) and serotonin and norepinephrine reuptake inhibitors (SNRIs) enhance medication adherence following access to a mobile application (app) tailored for this patient group. The study addresses the implementation phase of medication adherence.

**Methods:**

The study was a single group pre-post intervention design. Data were collected using the validated OsloMet Adherence-to-medication Survey tool (OMAS-37) before and after app access. Pre-app access survey (Survey 1) was conducted via social media and online newspapers, encompassing 445 SSRI/SNRI users aged 18 years and above. Post-app access survey (Survey 2) was sent to 103 SSRI/SNRI users from Survey 1. Wilcoxon Signed Rank Test compared pre- and post-intervention adherence measurements. Pearson’s chi-square tests and Fisher’s exact tests compared study population categories.

**Results:**

Forty-two SSRI/SNRI users, median age 26 (IQR 17), 93% identifying as female, used the app while using the same antidepressant during the 2-month period between gaining access to the app and Survey 2. There was a statistically significant reduction in non-adherence score post-app access (z = 3.57, n = 42, *p* < 0.001) with medium effect size (r = 0.39), indicating enhanced adherence. Total non-adherence score decreased by 39% from pre-to post-access, and there was a 12% decrease in users scoring equivalent with poor adherence (score <2) post-access. Twenty-nine of 37 non-adherence causes improved, with three showing statistical significance. Of 42 responders, 50% (n = 21) indicated using the app one to two times, while 50% (n = 21) more than three times. Approximately 69% (n = 28) found it useful, and 43% (n = 18) felt safer in their use of antidepressants after access to the app. No significant preference was observed for the app over alternative sources of information.

**Discussion:**

Enhanced medication adherence was observed among antidepressant users following access to the tailored app. Further studies are warranted to evaluate the app applicability to a broader range of antidepressants users or other patient groups, encompassing those in the initiation phase of medication adherence. The app is intended as an easily accessible supplement to the information and advice provided by prescribing physicians and dispensing pharmacists.

## 1 Introduction

Patients’ adherence to medication is often inadequate (World Health Organization, 2003; Foley et al., 2021), and there are numerous studies concerning factors impacting medication adherence (Cheen et al., 2019; Gast and Mathes, 2019). Reasons for non-adherence are complex and vary both among individual patients and within each patient over time (World Health Organization, 2003; Horne et al., 2019). For instance, these variations could be dependent on whether the medication regimen has recently been initiated or has been in place for an extended period, and the rationale behind a patient’s use of the medication (Horne et al., 2019). Given the influential effect of how long a patient has used a medication, the definition of medication adherence consists of three phases: the initiation phase (from prescribing to first dose), implementation phase (from first dose to discontinuation), and discontinuation (last dose). Alternatively, the initiation phase, implementation phase, and persistence phase - wherein persistence refers to the duration between the initiation and the last dose, which immediately precedes discontinuation (Vrijens et al., 2012; De Geest et al., 2018). In a clinical setting with patients who are non-adherent, it is crucial to ascertain the reasons for non-adherence, including the rationale behind a patient’s medication use, and to devise interventions accordingly. In their evidence-based guideline on medication adherence (2023), the National Institute for Health and Care Excellence (NICE) in the UK designates a key principle for interventions. According to NICE, while the improvement of adherence is feasible, there is no one-size-fits-all intervention suitable for every patient. The institute strongly advocates for personalized interventions tailored to address the unique adherence challenges faced by each individual (National Institute for Health and Care Excellence, 2019). Although individual adherence interventions are considered ideal, it might be more cost effective with interventions tailored for specific patient groups. Such an intervention could be used as an adherence-enhancing tool, for instance as a part of individual intervention guidance by health personnel. In this way, it could serve as a resource-saving measure, providing healthcare personnel with a quality-assured tool to offer specific patient groups as a supplement to individual counseling. In our previous study utilizing the validated OsloMet Adherence-to-medication Survey tool, OMAS-37, we revealed that patients using medication for Mental health disorders (MHD) were among the most non-adherent. This finding is supported by previous studies reporting poor adherence to medication for MHD-patients (Semahegn et al., 2020; Lassen et al., 2024). These patients could benefit significantly if such a tailored intervention provided an effect on adherence. The benefits include improved quality of life due to improved treatment outcomes and a reduced burden from adverse drug reactions. In our previous study we found that the five main causes of non-adherence for this patient group were “Forgot to take the medication”, “Having used the same type of medication before without them having good/satisfactory effect”, “Feeling better”, “Fearing adverse drug reactions” and “Having difficulties taking the medication to specific hours”. Therefore, addressing these causes would be important topics in a tailored intervention for this patient group. To be able to provide tailored information on for instance adverse drug reactions, medications for mental health disorders need to be narrowed down to a specific medication group. Among the psychiatric disorders, depression is the leading cause of disability (Abate et al., 2018). The recommended first-line pharmacological treatment for depression is second generation antidepressants, where selective serotonin reuptake inhibitors (SSRIs) and serotonin and norepinephrine reuptake inhibitors (SNRIs) are widely used (Kennedy et al., 2016; Qaseem et al., 2016; National Institute for Health and Care Excellence, 2022). According to the Norwegian National Institute of Public Health (NIPH), 7% of the population was prescribed at least one antidepressant in 2021. The proportion was higher among women and the older population: in 2021, 9% of women and 11% of those over 65 years old were prescribed at least one antidepressant (NIPH, 2022). An intervention tailored to enhance adherence among SSRI/SNRI users could consequently have a significant impact on a large number of individuals. Developing this intervention as a mobile application, referred to as app, would facilitate easy accessibility as smartphones are ubiquitous. Although previous systematic reviews display mixed evidence regarding the benefits of mobile health (mHealth) interventions on adherence to medication, over all these interventions seem to be beneficial (Anglada-Martinez et al., 2015; Hamine et al., 2015). The aim of this study was to examine whether access to an app specifically tailored for this patient group would improve self-reported medication adherence among patients using SSRI/SNRI medication.

## 2 Materials and methods

### 2.1 Study design and data collection

The study was designed as a single group pre-post intervention study. The data were collected using two OMAS-37-incorporated e-surveys: Survey 1 was conducted prior to the respondents’ access to an app, followed by Survey 2 conducted approximately 2 months after they gained access to the app. The study addressed individuals that were actively using medication, indication their presence in the implementation phase of adherence to medication (De Geest et al., 2018).

#### 2.1.1 The OMAS-37 incorporated e-surveys: Survey 1 and Survey 2

OMAS-37 is a validated adherence assessment tool (Larsen et al., 2022) that comprises 37 causes of non-adherence. The tool enables the calculation of individual responders’ non-adherence score on a scale from 0 to 111, with an increasing score indicating reduced adherence. Additionally, the tool facilitates the calculation of cause-specific adherence scores, providing insight into the most prominent causes for non-adherence. Alongside OMAS-37, both e-surveys encompassed questions regarding demographics and medication usage. In addition, respondents were asked to indicate the medical conditions for which they received medication within the last 12 months. The respondents were given the choice to select one or more medical-condition groups from a list of 24 choices, selected from The Norwegian Medicines Manual for Health Personnel (NMM, 2023) in addition to the options “other” and “do not know/do not want to tell/not applicable”. Predefined inclusion criteria for Survey 1 were using medication and being 18 years or older. Incorporated adaptive features excluded respondents not meeting the predefined inclusion criteria. Furthermore, the adaptive features of Survey 1 allowed users who checked off using medication for mental health disorders to be asked whether they used antidepressants for the treatment of depression. Those who responded affirmatively were further questioned about the specific type they used and could choose from a list of marketed SSRI/SNRI medications in Norway in addition to the options “none of these” and “do not know/do not want to tell/not applicable”. Those who reported using medication from this medication list were asked if they were interested in testing an app and provided with information about the study. Those who agreed were prompted to input their email addresses for further contact regarding consent and access to the app. To ensure anonymity, the responses to Survey 1 were sent directly to and stored in a secure server for sensitive data, TSD - Services for sensitive data (University of Oslo, 2024). Survey 2 closely resembled Survey 1, with the exception of the questions: whether they used antidepressants, if they wanted to participate in the study, or about leaving their email address. In Survey 2, the first question asked the respondents to provide a unique code they received in the same email as the online hyperlink to Survey 2. This was done to enable pairing of each respondent’s pre- and post-access responses without asking for personal information. Respondents in Survey 2 were also asked whether they were still using the same antidepressant as they did approximately 2 months ago, to ensure that they still belonged to the target group. Questions regarding the usage and usefulness of the app were also added, including one question comparing the app to information provided by sources other than a physician or pharmacist. Participants were asked to rate on a scale from 1 to 10, where 1 indicates a strong preference for other methods of obtaining information about medications (excluding physicians and pharmacists), and 10 indicates a strong preference for a quality-assured app like the one they were given access to. To ensure completeness of responses in both Survey 1 and Survey 2, all checkbox questions were mandatory. Prior to submission, respondents were afforded the option to navigate back to prior pages by selecting “Previous page”.

#### 2.1.2 The app

A web app was made specifically for the study in the non-code app builder Glide (Glide, 2023). Access was given by sending the respondents the app hyperlink/QR-code via email. The app content was tailored for individuals who had been prescribed SSRI/SNRI for depression by a physician and were actively using the medication. The content was developed based on the five main causes of non-adherence for users of medication for Mental Health Disorders identified in our previous study. An illustration depicting the content of the app can be found in the Supplementary Material. The information was given in accordance with Norwegian guidelines from the National Online Portal for Health Services in Norway and the Norwegian Directorate of Health. Quality-assured information was provided by entities like the national network of four regional medicines information and pharmacovigilance centers in Norway and the Norwegian Pharmaceutical Product Compendium. The information was conveyed in a manner designed to motivate good adherence and instill greater confidence regarding medication use. The app content was quality-assured by a resource group consisting of a physician (specialization in psychiatry and clinical pharmacology), a postdoctoral psychologist, and four pharmacists with clinical experience: a professor, two associate professors, and a post doctor scientist. Additionally, the resource group included three members from the intended target group, one female and two males. The app underwent three successive releases for quality assurance by the resource group, where each version was reviewed, revised, and then followed by release and subsequent review of a new iteration. After the third version, no major comments were made. Following the revision, the resource group evaluated the app by employing the user version of the mobile Application Rating (uMARS) (Stoyanov et al., 2016) to assess the app. Based on uMARS the app quality mean score was 4.3 out of 5. Response rate was 78%, which included the three members from the target group. The app was ultimately named ADA (AntiDepressantsApp).

#### 2.1.3 Data collection

Recruitment for Survey 1 was carried out by distributing invitations, each containing a hyperlink to Survey 1, through social media and 15 online newspapers during July to October 2023. On Facebook, the invitation was directed to individuals living in Norway, aged 18 years or older and using medication. The invitation was posted on one of the researcher’s Facebook-page and Messenger-account with encouragement to share the invitation. Furthermore, the invitation was posted on large Norwegian Facebook groups that encompassed both health related and non-health related subjects. Paid advertising was used on Snapchat, in 14 online newspapers and in an online magazine for the Mental Health Council, to recruit adults aged 18 or older who used antidepressants.

All communication with participants who provided their email addresses in Survey 1 occurred through a dedicated study email account. This communication included providing study information, seeking, and receiving consent, delivering the app access hyperlink/QR-code and hyperlink to Survey 2. In addition, approximately 1 week after the app hyperlink/QR-code was sent, the recipients were sent an email reminder to utilize it. The hyperlink to Survey 2 was sent around 2 months after delivering app access. The data from Survey 2 were collected from September 2023-January 2024.

### 2.2 Sample size determination

Sample size was calculated based on a two-sided paired *t*-test, using a 5% significance level, 80% power and a standard deviation (SD) of 9. SD was based on our previous OMAS-37 study. To detect a change of 3.5 points using a paired design, 62 participants were needed.

### 2.3 Statistical methods

General features of the study population were described using medians, interquartile range (IQR), numbers and percentages. SSRI/SNRI users were divided into those who did not want to use the app, referred to as non-app users, and those who did use the app and continued to use the same antidepressant after 2 months, referred to as app users. Predefined groups of categorical features were compared between the non-app users and app-users using Pearson’s chi-square test. Fisher’s exact tests were performed when the sample size of the selected groups was too low for Pearson’s chi-square tests. Pre- and post-intervention adherence measurements were compared using the non-parametric Wilcoxon Signed Rank Test. The same test was used *post hoc* for comparing pre- and post-non-adherence scores for each of the specific causes of non-adherence. Despite performing power calculations based on a parametric test (paired *t*-test), we decided to perform a non-parametric test comparing pre- and post-intervention measurements since the conditions of the *t*-test were not fulfilled (normal assumption). OMAS-37 differentiates between a statistical cut-off score for good versus poor adherence by a threshold of 10 points, and a clinical cut-off score by a threshold of two points (Larsen et al., 2022). In this study, the clinical cut-off score of two was utilized, suggesting that a score of 1or 0 indicates good adherence. All data were analyzed by SPSS Statistics version 27, R version 4.3.0, and Microsoft 365 Excel version 2,208. The selected significance level alpha was 0.05. The results are reported in accordance with the ESPACOMP Medication Adherence Reporting Guideline (EMERGE) (De Geest et al., 2018).

## 3 Results

### 3.1 Selection and demographic characteristics of respondents

#### 3.1.1 Demographic and medication profile

**FIGURE 1:**
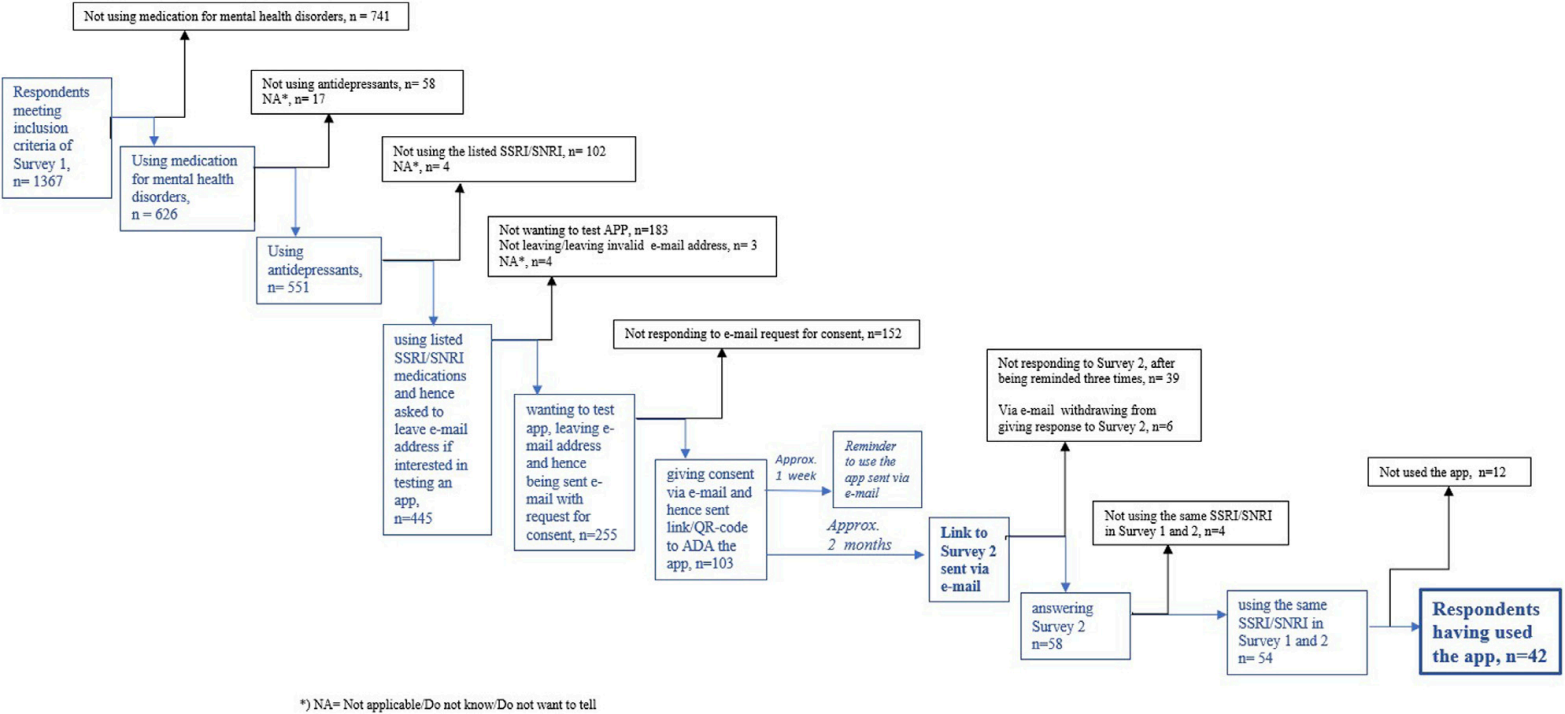
Respondents’ flowchart.

Survey 1 was answered by 1,367 respondents, of whom 445 indicated that they were using SSRI/SNRIs and were therefore asked to participate in the subsequent study (Figure 1). A total of 103 respondents gave their consent and were given access to the app. Approximately 2 months later, these respondents were sent Survey 2. Of the 58 respondents who completed Suvery 2, 42 were using the same SSRI/SNRI medication during the approximatley 2-month period between answering the two surveys, while also using the app. The demographics (Table 1) revealed that the 445 respondents who reported using SSRI/SNRIs were significantly younger, with a median age of 26 years (IQR 17), compared to the total sample of 1,367, which had a median age of 47 years (IQR 31). The subgroup of SSRI/SNRI users who were using the app (n = 42), had the same median age as the SSRI/SNRI users who did not use the app (n = 403). Male participation was low across all three groups, with 15% for the total sample, 10% for the non-app users, and 5% for the app users.

**TABLE 1:** Demographics of the total respondents of Survey 1, the SSRI/SNRIs group not using the app, and SSRI/SNRI group using the app.

Sample		Total *n* = 1367 (100%)	Using listed SSRI/SNRI and not app, *n* = 403 (100%)	Using listed SSRI/SNRI and app, *n* = 42 (100%)
Age in years	Median	47.0	26.0	26.0
	IQR	31.0	17.0	17.0
	Young 18–44 years	647 (47)	330 (82)	39 (93)
	Middle aged 45–65 years	535 (39)	56 (14)	3 (7)
	Young-elderly 66–79 years	163 (12)	15 (4)	0 (0)
	Elderly-Elderly 80–89 years	22 (2)	2 (<1)	0 (0)
Gender	Female	1141 (84)	353 (88)	39 (93)
	Male	206 (15)	41 (10)	2 (5)
	Other	16 (1)	9 (2)	1 (2)
	NA*	4 (<1)	0 (0)	0 (0)
Education level	K-12 education		277 (69)	30 (71)
	Postsecondary education		120 (30)	12 (29)
	NA**		6 (1)	0 (0)
Number of selected medical condition groups	1–2		153 (38)	12 (29)
	3–4		138 (34)	14 (33)
	5 or more		112 (28)	16 (38)
Years of regular medication use	0–1		49 (12)	5 (12)
	2–5		126 (31)	16 (38)
	6 or more		225 (56)	16 (50)
	NA*		3 (1)	0 (0)
Number of daily medications	1–2		203 (50)	21 (50)
	3 or more		197 (49)	21 (50)
	NA*		3 (1)	0 (0)
“Anchor question”: To what extent they believe they are following the recommendations from their doctor regarding their medication use	To a very large extent		259 (64)	29 (69)
	To a large extent		123 (31)	12 (29)
	To a limited extent		12 (3)	1 (2)
	To a very limited extent		4 (1)	0 (0)
	NA*		5 (1)	1 (2)
Utilizing pill organizer and/or pre-packed medicine	Yes		184 (46)	21 (50)
	No		214 (53)	21 (50)
	NA*		5 (1)	0 (0)

There were no statistical differences between the app-users and the non-app users in education, number of medical conditions or medication usage. A greater proportion of the non-app users (38%) had one to two conditions compared to the app users (29%), and a greater proportion of the app users (38%) had more than five conditions compared to the non-app users (28%).

#### 3.1.2 Recruitment of app users

**FIGURE 2:**
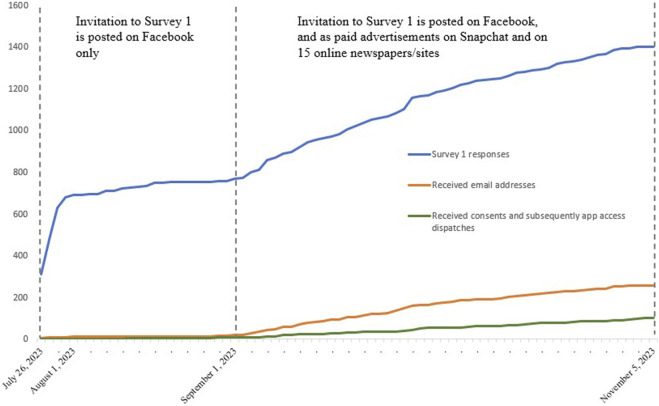
Timeline for Survey 1 recruitment and app access dispatches.

The timeline of Survey 1 recruitment, received email addresses, consents, and app access dispatches is shown in Figure 2. The final participant received app access on 5 November 2023.

### 3.2 Adherence measurements

Among the app users, 60% (n = 25) had a decrease in non-adherence scores indicating enhanced adherence following access to the app. There was an observed decrease of approximately 39% in the total non-adherence score. A Wilcoxon Signed Rank Test revealed a statistically significant reduction in non-adherence scores, z = 3.57, n = 42, *p* < 0.001, with a medium effect size (r = 0.39). The median adherence score decreased from 8.5 pre-access to 5.2 post access, showing better adherence to medication after access to the app. Before access to the app 79% (n = 33) exhibited non-adherence scores from two points and above, indicating poor adherence. The highest score was 41 points, exhibited by one respondent. After access to the app 67% (n = 28) exhibited scores from two points and above. The highest score was then 25 points, exhibited by one respondent. Of the 40% who did not experience a decrease in non-adherence points following app access, 26% (n = 11) exhibited an increase, suggesting poorer adherence after accessing the app, while 14% showed no change in non-adherence scores before and after accessing the app.

### 3.3 Changes in the non-adherence score for the 37 causes of non-adherence

Table 2 displays the pre- and post-non-adherence scores for all the 37 causes of non-adherence sorted by highest total non-adherence score pre-access to app. Twenty-nine of the causes had a decrease in non-adherence scoring, meaning improved adherence. The largest changes were the decrease in 10 points for each of the causes “Taking medication is a reminder of being ill” and “Having difficulties taking medication due to specific instructions (e.g., with and without food, in upright position, etc.)”. The *post hoc* comparison of the pre- and post-access scores for each cause individually revealed statistically significant differences in median score for the three causes “Taking medication is a reminder of being ill” (*p* = 0.02), “The medication has not had noticeable effect” (*p* = 0.01), and “Need to be able to drive a car” (*p* = 0.04). The causes “Forgot to take the medication” and “Having difficulties taking the medication to specific hours” were the most common causes for non-adherence both pre- and post-access to the app.

**TABLE 2:** Pre- and post-access non-adherence scores and changes for the 37 causes of non-adherence. Arranged by highest total non-adherence score pre-access. *) Statistically significant changes.

Causes of non-adherence	Total non-adherence score pre-access to the app	Total non-adherence score post-access to the app	Difference in total non-adherence score pre-and post-access to the app
Forgot to take the medication	34	30	4
Having difficulties taking the medication to specific hours	32	24	8
Taking medication is a reminder of being ill	21	11	10 (*p* = 0.02)*
Cannot stand taking medication	19	16	3
Having difficulties taking medication due to specific instructions (e.g., with/without food, in upright position, etc.)	19	9	10
The medication was sold out/unavailable at the pharmacy	17	10	7
Feel stigmatized or ill by having to use the medication	14	11	3
Do not want others to know that I am taking medication	14	11	3
Feeling better	14	9	5
Financial reasons	14	7	7
Have no medication left	13	9	4
Fear of adverse drug reactions	13	5	8
Using many drugs simultaneously	13	4	9
Do not feel ill	11	6	5
The same type of medication has been used before without having good/satisfactory effect	11	5	6
Feel worse when taking the medication	11	2	9
The medication has not had noticeable effect	11	2	9 (*p* = 0.01)*
Reckon makes no differences whether using the medication or not	9	3	6
Incompatible with lifestyle	8	10	−2
Fear of becoming addicted to the medication	8	4	4
Feel clever when taking less than recommended by the physician	8	3	5
Need to be able to drive a car	8	3	5 (*p* = 0.04)*
Belief the medication is harmful/toxic, and/or cannot tolerate it	7	5	2
Am against medication as a matter of principle	5	4	1
Prefer alternative treatment	5	4	1
Difficulties accessing a pharmacy	4	2	2
Misunderstandings related to generic medication (medication with same content but from different manufacturers)	3	3	0
Practical reasons (e.g., difficulty opening packaging or pressing tablets out of blister packs, or splitting/crushing the tablets)	3	2	1
Difficulties taking the medication due to disability or impaired vision	3	0	3
Little or no information from physician/pharmacy/other health personnel about how to use the medication	2	2	0
Being pregnant	2	1	1
Influenced by media/internet/friends/family/others	2	1	1
Did not understand the physician/pharmacist’s instructions	0	1	−1
Breastfeeding	0	1	−1
Forgot how to take it	0	0	0
Ethical/religious reasons	0	0	0
Reluctance to visit a pharmacy due to the corona pandemic	0	0	0

### 3.4 App use and evaluation

Among the 42 respondents 50% reported having utilized the app one to two times, 38% reported having used it three to five times, and 12% reported having used it more than five times. More than two-thirds (69%) of the respondents found the app useful, and 43% reported an increase in confidence in their usage of antidepressants after gaining access to the app (Table 3). The respondents were asked to rate the statement, “Think about the ways you gather information about the medications you use (excluding information from physicians and pharmacies) and compare these with the app”. They were asked to provide a rating between one and ten, with one representing reliance solely on other sources, and ten indicating reliance solely on the app. The median score for this statement was found to be 7 (IQR 4), n = 42. A Wilcoxon Signed Rank Test did not give a significant result (*p* = 0.14), indicating that there was no significant preference for the app over other sources of information (scoring 5.5 and above). The app had no reminder function, so the respondents were also asked whether they were using any reminders on their mobile phone: 12% were using another reminder app, 48% used the reminder function of the mobile phone, and 40% did not use any reminder on the mobile phone.

**TABLE 3:** Respondents’ utilization and evaluation of the app.

Questions		*n* = 42 (100%)
How frequently would you estimate you have utilized the app?	1–2 times	21 (50)
	3–5 times	16 (38)
	More than five times	5 (12)
How would you assess the usefulness of the app for your needs?	No usefulness	13 (31)
	Some usefulness	13 (31)
	Moderate usefulness	10 (24)
	Great usefulness	6 (14)
Has your confidence in using antidepressants increased after accessing the app?	No difference	24 (57)
	Somewhat safer	13 (31)
	Much safer	5 (12)

## 4 Discussion

There are many apps for mental health disorders like depression, but few of these seem to be supported by solid scientific evidence, regarding both evidence-based guidelines and statistically significant adherence improvements (Wasil et al., 2019; Ng et al., 2020; Eis et al., 2022). In addition, many apps attempt to cover too many topics, resulting in limited and insufficient information on each topic (Martinengo et al., 2022). In this study the app was tailored for SSRI/SNRI medication users based on our previous findings of main causes of non-adherence for this group. A statistically significant reduction of non-adherence score following access to the app was found, with a medium effect size - indicating improvement in adherence to medication after access to this app. The non-adherence scores decreased for 60% of the app users. Tailoring an app for specific medication users based on main causes of non-adherence presents a new approach in enhancing adherence to medication. This would be beneficial especially for patient groups that are facing numerous challenges with adherence and treatment, such as those using antidepressants.

### 4.1 Main findings and clinical implications

Improving adherence to antidepressants can enhance patients’ quality of life and diminish resource use in the healthcare system (Ta et al., 2021). However, patients’ reasons for non-adherence are complex (World Health Organization, 2003; Horne et al., 2019) and when developing interventions, it is important to address the main causes for whom the intervention is designed. This study has tailored an app for SSRI/SNRI medication users, after the main causes of non-adherence for this group were identified by using OMAS-37. Access to the app resulted in a statistically significant reduction in non-adherence scores, and a 12 percentage-point decrease in number of app users that scored equivalent with poor adherence. The most common non-adherence cause, both before and after access to the app, was “Forgot to take the medication”. This cause experienced a decrease of only 12%. This was anticipated, as although this issue was addressed in the app, a duration of 2 months is typically too short a duration to alter habits. There was no significant change in use of pill organizer pre- and post-access to the app. Non-adherence was improved in 29 of the 37 causes of non-adherence, where statistical significance was found for three of the causes, all three of which were directly addressed in the app. Although this was an explorative *post hoc* analysis, these results could be useful in future studies for generating hypotheses regarding the app-effect on individual causes. The main comments from the 12 respondents that had access to the app and did not use it, and the 10 respondents answering “Other” to the question “Why was the app of no or only little use to you?” - was that they already had knowledge of the information given in the app. The app users rated their reliance on the app compared to other sources of information (besides information given by physicians and pharmacies). The results showed a preference for the app, although not statistically significant. There were no significant demographic differences between the non-app users and the app users, suggesting that the app users are representative of SSRI/SNRI users. Given the vast number of individuals using SSRI/SNRI medication, it was expected that there would be variation in knowledge levels, and the app is tailored to those with a lower level of knowledge. Therefore, despite the fact that the app is designed for patients in the implementation phase of medication adherence–those actively using medication - it is plausible that not all users will discover new information within the app. The varied interest in accessing the app (58 out of 445) could likely be attributed as much to individual enthusiasm for testing new things as to challenges associated with the use of antidepressants. While only half of the app users felt more confident in their use of antidepressants after having accessed the app, two-thirds of the participants rated the app as useful to them. Given the number of overall antidepressant users, the potential availability of the app to a broader population could hold significant clinical implications, suggesting a substantial impact on a large number of patients. In Norway, between six and seven percent of females aged 20 to 25 used antidepressants in 2020, with a vast majority of them utilizing SSRIs (NIPH, 2024). Therefore, even if the app were to be exclusively used by young females, as many of the respondents in this study were, the app could have a significant impact. 

In addition, comparable app tailoring approaches may be implemented to assess their efficacy in improving medication adherence in other patient groups. Our findings are consistent with previous research that, although there is mixed evidence, regards mHealth interventions to be beneficial (Anglada-Martinez et al., 2015; Hamine et al., 2015).

### 4.2 Methodological considerations

It is reasonable to posit that the age discrepancy between the overall sample and the users of SSRI/SNRI medication may be attributed to the likelihood that Snapchat served as one of the platforms from which many app users likely were recruited. The paid advertisements on Snapchat and online newspapers/sites were much more effective in recruiting potential app users compared to postings on Facebook alone, as can be seen in Figure 1. In 2022, 86% of women and 82% of men aged between 18 and 29 years in Norway were using Snapchat, whereas it was less popular among those aged 50 years and older (Statista, 2023). A challenge was associated with the algorithms of specific social media platforms. We sought to avoid the algorithms from singling out individuals responding exclusively about antidepressant usage; instead, we extended the invitation to all medication users. Consequently, we anticipated that many respondents might not meet the inclusion criteria. As illustrated in Figure 1, out of the 1,367 respondents, only 445 met the inclusion criteria. Out of the 445 individuals meeting the inclusion criteria, only 255 expressed a willingness to participate in using the app. This reluctance could partly be due to the loss of anonymity upon participation. Among the 255 who provided their email addresses, only 40% responded with consent, a figure somewhat lower than anticipated. This lower response rate might be associated with less frequent usage of their private email accounts among young people, suggesting that utilizing social media for all of the correspondence could have been more effective (Janssen and Carradini, 2021). Among the 103 individuals who gave consent, 56% responded to Survey 2, a result in line with expectations for surveys of this nature.

The recruitment period was not long enough to recruit the number of participants (n = 62) necessary based on the power calculations. This was due to the given time frame. However, the final achieved sample of 42 was sufficient to show a significant reduction in non-adherence with medium effect size. Previous studies indicate that non-adherence increases over time of medication use (World Health Organization, 2003; Khan and Socha-Dietrich, 2018). The majority of participants (88%) had been using their medications for more than 2 years, and all had been using the SSRI/SNRI medication for at least the 2-month duration of the study. Despite this, a statistically significant increase in adherence was observed, which could further corroborate the effectiveness of the app. The hyperlink to Survey 2 was distributed 2 months after the participants gained access to the app. The trial period had to be long enough for changes to take place, but not so extensive that the participants would lose interest in the study. The app could primarily motivate change by providing information, advice, and guidance, and 2 months was considered long enough for motivational changes to take place.

The validated Mobile Application Rating Scale (MARS) (Terhorst et al., 2020) requires mobile mHealth knowledge and training. Consequently, the end-user version, uMARS, was chosen for the validation of the app by the resource group. Based on uMARS the App Quality Mean Score was 4.3 out of 5 from the resource group. The main reason for not scoring 5 was that the app did not have any interactivities or customizations, and thus scored very low in this section of uMARS. Interactivities or customizations can be created to a certain extent in Glide but were not developed in this project. Implementing these features in a web app could also interfere with the app users’ anonymity. A significant advantage in app development lies in the simultaneous testing of functionality alongside quality assurance for content. Consequently, the feasibility was quality assured by the resource group concurrently with content assurance.

### 4.3 Limitations

This study was a single group study, and in a single pre-post intervention design one cannot easily control for extraneous variables or determine causality. When conducting an intervention, the Hawthorne effect, which suggest that being examined in itself brings about behavioral changes, has to be taken into consideration. While this effect is commonly acknowledged, still little seems to be known about its mechanisms or magnitude (McCambridge et al., 2014). The effect size of accessing the app was medium, suggesting that this is likely not solely due to the Hawthorne effect. One potential bias is that while self-reporting is a frequently used method for assessing adherence due to its low cost, flexibility, discretion, and time efficiency, it tends to overestimate medication adherence. This overestimation can be attributed to social desirability bias, a phenomenon where individuals respond in a manner they believe will be viewed as socially acceptable or favorable, rather than providing responses that accurately reflect their true thoughts, feelings, or behaviors (Lehmann et al., 2014). Nevertheless, since this bias is presumed to be consistent in both pre- and post-surveys due to the parried test design, any changes in scores may not inherently reflect a bias. Recruitment via social media for patients using SSRI/SNRI could be less suitable for older patients. The median age for respondents in this study was 26 years IQR 17). In addition, literature indicates that women are more likely to seek health-related information online than men and are more inclined to respond to online inquiries (Smith, 2008; Bidmon and Terlutter, 2015; Wu et al., 2022). In this study 93% of the app users identified as women. Further studies are therefore required to determine whether the findings also apply for older adults and to men. Possible long-term effects on non-adherence could not be assessed, as the pre-post access part period of the study was restricted to 2 months.

### 4.4 Conclusion

This study describes the testing of a tailored app to enhance medication adherence for users of antidepressants, utilizing the OMAS-37 adherence assessment tool. Access to the app proved to enhance adherence to medication. This study is the first to use OMAS-37 as an adherence assessment tool for an intervention. Further studies are required to evaluate the applicability of the app to a broader range of antidepressants users, encompassing those in the initiation phase of medication adherence. Furthermore, to determine whether a comparable tailoring approach can be applied to other patient groups. The app is intended as an easily accessible supplement to the information and advice provided by prescribing physicians and dispensing pharmacists.

## Data Availability

The raw data supporting the conclusion of this article will be made available by the authors, without undue reservation.
