# Supplementary material for: Access to a tailored mobile application enhances medication adherence among young users of antidepressants
Source: Front Pharmacol. 2024 Apr 10;15:1379700. doi: 10.3389/fphar.2024.1379700 (PMC11039783; doi:10.3389/fphar.2024.1379700)
Supplement: Supplementary file 1 [file Presentation1.pdf]

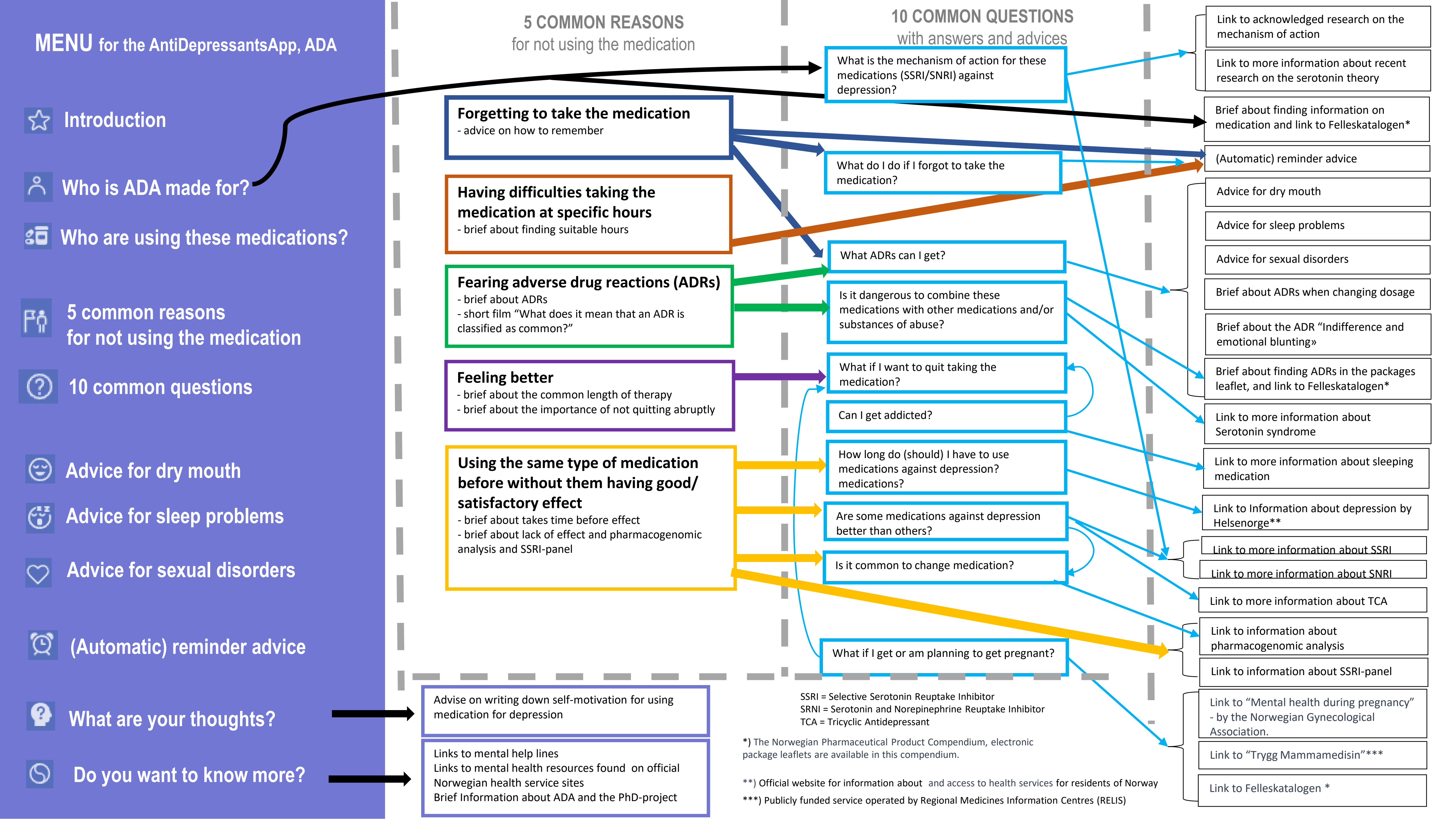
MENU for the AntiDepressantsApp, ADA
Introduction
Who is ADA made for?
Who are using these medications?
5 common reasons for not using the medication
10 common questions
Advice for dry mouth
Advice for sleep problems
Advice for sexual disorders
(Automatic) reminder advice
What are your thoughts?
Do you want to know more?
5 COMMON REASONS for not using the medication
Forgetting to take the medication
- advice on how to remember
Having difficulties taking the medication at specific hours
- brief about finding suitable hours
Fearing adverse drug reactions (ADRs)
- brief about ADRs
- short film "What does it mean that an ADR is classified as common?"
Feeling better
- brief about the common length of therapy
- brief about the importance of not quitting abruptly
Using the same type of medication before without them having good/ satisfactory effect
- brief about takes time before effect
- brief about lack of effect and pharmacogenomic analysis and SSRI-panel
10 COMMON QUESTIONS with answers and advices
What is the mechanism of action for these medications (SSRI/SNRI) against depression?
What do I do if I forgot to take the medication?
What ADRs can I get?
Is it dangerous to combine these medications with other medications and/or substances of abuse?
What if I want to quit taking the medication?
Can I get addicted?
How long do (should) I have to use medications against depression? medications?
Are some medications against depression better than others?
Is it common to change medication?
What if I get or am planning to get pregnant?
Link to acknowledged research on the mechanism of action
Link to more information about recent research on the serotonin theory
Brief about finding information on medication and link to Felleskatalogen*
(Automatic) reminder advice
Advice for dry mouth
Advice for sleep problems
Advice for sexual disorders
Brief about ADRs when changing dosage
Brief about the ADR "Indifference and emotional blunting»
Brief about finding ADRs in the packages leaflet, and link to Felleskatalogen*
Link to more information about Serotonin syndrome
Link to more information about sleeping medication
Link to Information about depression by Helsenorge**
Link to more information about SSRI
Link to more information about SNRI
Link to more information about TCA
Link to information about pharmacogenomic analysis
Link to information about SSRI-panel
Link to "Mental health during pregnancy" - by the Norwegian Gynecological Association.
Link to "Trygg Mammamedisin"***
Link to Felleskatalogen *
Advise on writing down self-motivation for using medication for depression
Links to mental help lines
Links to mental health resources found on official Norwegian health service sites
Brief Information about ADA and the PhD-project
SSRI = Selective Serotonin Reuptake Inhibitor
SRNI = Serotonin and Norepinephrine Reuptake Inhibitor
TCA = Tricyclic Antidepressant
*) The Norwegian Pharmaceutical Product Compendium, electronic package leaflets are available in this compendium.
**) Official website for information about and access to health services for residents of Norway
***) Publicly funded service operated by Regional Medicines Information Centres (RELIS)
